# Correlation of physiological stress as measured by heart rate and blood pressure variability and self-reported anxiety

**DOI:** 10.21203/rs.3.rs-6941070/v1

**Published:** 2025-09-17

**Authors:** Gurjeet Birdee, Hui Nian, Sachin Paranjape, Robert Abraham, Andre Diedrich, Kerry Kinney, Alfredo Gamboa

**Affiliations:** Vanderbilt University Medical Center; Vanderbilt University Medical Center; VUMC: Vanderbilt University Medical Center; VUMC: Vanderbilt University Medical Center; VUMC: Vanderbilt University Medical Center; VUMC: Vanderbilt University Medical Center; VUMC: Vanderbilt University Medical Center

## Abstract

**Background:**

Despite lack of data, there is an assumption that anxiety symptoms correspond with a physiological stress response with decreased parasympathetic and increased sympathetic nervous system.

**Objective:**

To examine whether self-reported measures of anxiety correlate with physiological stress.

**Methods:**

We performed a secondary analysis of data collected from a 12-week clinical trial on slow breathing that examined the changes in psychological and physiological stress (n = 88). Psychological stress was assessed with PROMIS Anxiety CAT. Physiological stress was assessed by spectral analysis of heart rate including RMSSD, LF_RRI_, HF_RRI_, LF_RRI_/HF_RRI_ ratio and blood pressure variability (LF_sys_). Analyses consisted of Spearman correlations calculated at specific time points (baseline, 6, and 12 weeks) and changes over time (6 weeks vs. baseline, 12 weeks vs baseline) between PROMIS anxiety and physiological stress measures. Also, linear mixed-effects models were fitted on PROMIS Anxiety for physiological measures.

**Results:**

Despite a decrease in mean PROMIS Anxiety from baseline to 12 weeks (−4.85 S.D. ± 5.53) from slow breathing, we found a low correlation between PROMIS Anxiety and physiological measures of stress for specific time points or changes over time. Measures of autonomic tone, including LF_RRI_, HF_RRI_, LF_RRI_/HF_RRI_ ratio and LF_SYS_ showed poor correlation to PROMIS anxiety measures at baseline, 6, and 12 weeks. This suggests a disconnect between self-reported anxiety and indexes of autonomic tone.

**Conclusion:**

We report a lack of correlation between self-reported anxiety and physiological measures of stress despite reductions in self-reported stress after a behavioral intervention for relaxation.

## Introduction

The most common mental health disorders in the world are anxiety disorders.^1^ Anxiety significantly impacts mental and physical health, causing emotional distress, reduced quality of life, impaired social and occupational functioning,^2^ and a heightened risk of comorbid conditions like depression.^3^ Furthermore, anxiety disorders are linked to various physical health issues, including cardiovascular disease,^4^ gastrointestinal problems,^5^ and chronic pain.^6^ This substantial burden highlights the need to address anxiety as a public health priority.

Anxiety often presents as an excessive response to stressor that exceeds an individual’s coping abilities.^7^ Psychological symptoms of anxiety may present such as fear and nervousness, worry, and/or a sense of doom.^8^ Anxiety may also present with physical symptoms such as tachycardia, dry mouth, increased muscle tension, dizziness, and/or insomnia^8^. Physical symptoms match a physiological response to stress orchestrated by the autonomic nervous system including increased sympathetic and reduced parasympathetic tone (commonly referred to as “fight or flight” response). For individuals with anxiety disorders, studies suggest a correlation with increased sympathetic tone. Oppositely, a relaxation response has been reported to correlate with decreased sympathetic tone and increased parasympathetic tone.^9^ By measuring spectral analysis of heart rate variability (HRV) and blood pressure variability (BPV) we can assess different measures of sympathetic and parasympathetic tone as described in Table 1.

We have previously conducted a clinical trial among healthy adults (N = 99) to examine the effects of 12 weeks of slow breathing exercises on psychological and physiological stress.^10^ Slow breathing is a very common technique used to reduce anxiety symptoms. In this study, we measured psychological stress at baseline, 6 weeks, and 12 weeks with PROMIS Anxiety, observing a significant decrease in subject reported anxiety over time. We did not observe significant changes in physiological stress as measured by spectral analysis of HRV. Participants demonstrated a high adherence to slow breathing treatments with a mean class attendance of 10.7 out of 12 available sessions and an average of 4.8 home practices per week during the 12 weeks.

The purpose of this report is to examine whether self-reported measures of anxiety correlate with physiological stress as measured by spectral analysis of heart rate and BPV. Based on current literature, we hypothesized: 1) Higher psychological anxiety would be associated with higher sympathetic and lower parasympathetic tone as measured by spectral analysis in cross-sectional data analyses, and 2) Changes in psychological stress would be associated with decreases in sympathetic and increases in parasympathetic tone as measured with spectral analysis of heart rate and BPV from baseline to 12 weeks.

## Methods

The study was approved by the Vanderbilt University Institutional Review Board. The study methods have been previously published in detail.^10^ In summary, we enrolled healthy adults 30–60 years old in a single-arm study where participants received 12 weeks of slow breathing instruction on a weekly basis with a yoga teacher. Participants were also asked to perform breathing exercises daily for 12 weeks. Baseline, 6-, and 12-week measures of self-reported anxiety with PROMIS-29 and physiological stress with HRV and BPV were assessed. Among 99 participants, 93 completed both psychological and physiological data assessments for baseline, 6 weeks, and 12 weeks. 88 participants had complete data on all measurements and were included in the analyses. PROMIS Anxiety computerized adaptive test (CAT) is a 29-item instrument that has been validated as an anxiety measure in healthy adults. HRV was assessed in the Vanderbilt Autonomic Dysfunction Center as per standard protocol and assessment for autonomic testing.^11^ Participants were instructed to fast 8 hours and hold any medications and supplements that affect autonomic tone before assessments. All subjects were assessed in the morning with quiet rest in the supine position for at least 30 minutes prior to measurements. Blood pressure and heart rate were determined continuously using the finger clamp method (Nexfin, BMEYE, Amsterdam, the Netherlands) and ECG (VitalGuard450C, Ivy Biomedical Systems, Inc., Branford, CT). Cardiovascular signals were digitized (16 bit, 1 kHz) using a Windaq system (DI720; DATAQ Instruments, Akron, OH). Spectral analysis of heart rate and blood pressure variability was done following Task Force recommendations^11^ as described previously. Briefly, data segments of 300 seconds were recorded in supine subjects, and processed offline using own customized (A.D.) software “Physiowave” written in PV-Wave (Visual Numerics Inc., Boulder, CO) with power spectral densities estimated in low-frequency (0.04–0.15 Hz) and high-frequency (0.15–0.40 Hz) ranges using a fast Fourier transform–based Welch algorithm. Under circumstances of sympathetic activation, low frequency variability of systolic blood pressure (LF_sys_) is an indirect measure of sympathetic mediated modulation.^12^ High frequency variability of heart rate (HF_RRI_) measures cardiac parasympathetic modulation.^13,14^ The ratio of low frequency variability of heart rate (LF_RRI_) over HF_RRI_ (LF_RRI_:HF_RRI_) was calculated to access frequency dependent changes in cardio-vagal modulation.^11,13^ Table 1 provides the definition of anxiety, HRV, and BPV measures.

### Statistical Methods:

All the spectral measures of HRV and BPV are highly right-skewed and were log-10 transformed to make their distributions more symmetrical and closer to a normal distribution. The correlations between spectral measures and PROMIS Anxiety at each specific time point were assessed using Spearman correlation coefficients. Spearman correlation coefficients were also calculated for the change in HRV measures and change in PROMIS Anxiety from baseline to 6 and 12 weeks. Separate linear mixed-effects models were fitted for PROMIS Anxiety on each of spectral measures as well as age, gender and BMI, with a compound symmetry covariance matrix. Partial correlation was calculated using package LMMstar.^15,16^ All the analyses were performed using statistical software R 4.3.3 (R Core Team (2024), R: A Language and Environment for Statistical Computing. R Foundation for Statistical Computing, Vienna, Austria. https://www.R-project.org/).

## Results

**Figure 1:**
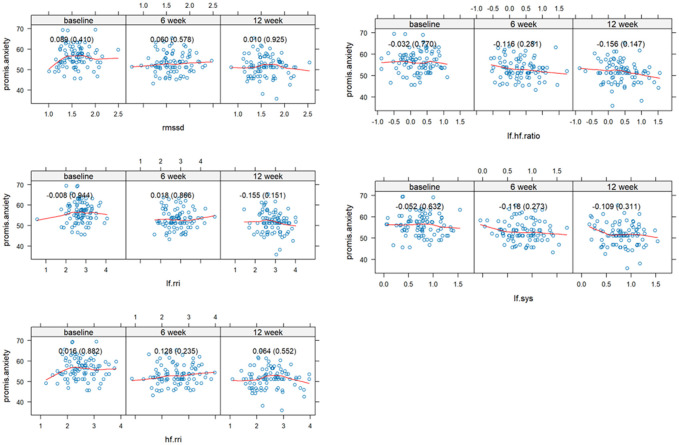
Spearman correlations between PROMIS Anxiety and HRV at baseline and 6- and 12-week assessments. All the HRV measures were log10 transformed. **Spearman** correlations between PROMIS Anxiety and spectral parameters at baseline and 6- and 12-week assessments. All spectral parameters other than RMSSD were log10 transformed. Each panel displays scatter plots with fitted trend lines showing the association between PROMIS Anxiety and spectral parameters. Spearman correlations and corresponding p-values are presented in parentheses within each panel.

**Figure 2:**
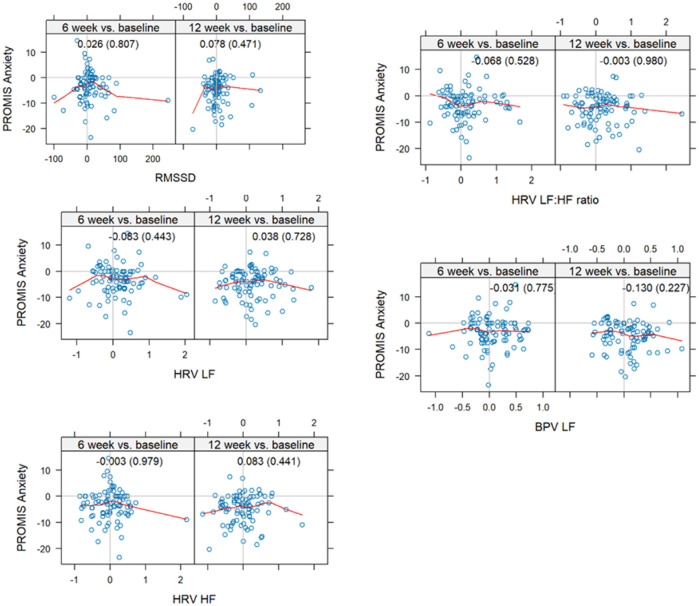
Correlations between HRV and BPV changes from baseline to post-randomization assessments and changes of PROMIS Anxiety Score. The changes of HRV measures at each time point. All the HRV measures were log10 transformed except RMSSD. **Spearman** correlations between HRV and LF_sys_ changes from baseline to post-randomization assessments. All spectral parameters other than RMSSD were log10 transformed. Scatter plots show the relationships between change in PROMIS Anxiety and spectral parameters from baseline to 6 weeks and 12 weeks. All change scores reflect difference from baseline. Spearman correlation coefficients and p-values are shown in parentheses within each panel.

The study sample consisted of 76% females with a mean age of 41.6 ± 8.9 years and 80% were white with a mean body mass index of 25.8 ± 5.1. Measures of autonomic modulation including HF_RRI_, LF_RRI_, LF_RRI_/HF_RRI_ showed poor correlation to PROMIS anxiety measures at baseline, 6, and 12 weeks (Fig. 1). This suggests a disconnect between self-reported anxiety and indexes of autonomic tone. The highest correlations were observed at 6 weeks for HF_RRI_ at 0.128, 12 weeks, LF_RRI_ at − 0.155, and 12 weeks for LF_RRI_/HF_RRI_ at − 0.156. We previously reported a significant decrease in mean PROMIS Anxiety from baseline to 12 weeks (−4.85 S.D. ± 5.53) from slow breathing.^10^ Here we report a lack of correlation between changes in autonomic tone and changes in PROMIS anxiety from baseline to 6- and 12-week assessments (Fig. 2). The highest correlation was − 0.130 (p = 0.026) for LF_sys_ which is clinically insignificant. This suggests changes in psychological stress from slow breathing are not related to changes in cardiovascular autonomic measures. A linear regression model adjusted for age, gender, and body mass index, showed low correlations for autonomic indexes (Table 2). Two measures, LF_RRI_/HF_RRI_ (rho: −0.170) and LF_sys_ (rho: -0.136), showed significant correlations, though LF_sys_ had a negative correlation with PROMIS Anxiety, which is counterintuitive, contradicted our original hypothesis, and clinically insignificant.

## Discussion

We examined the relationship of self-reported anxiety and spectral analysis of heart rate and blood pressure among healthy adults receiving a 12-week yoga-based slow breathing intervention. In this healthy population, longitudinal changes and cross-sectional data for self-reported anxiety did not correlate with changes in parasympathetic or sympathetic tone as measured through autonomic assessments. When adjusted for age, gender, and BMI, LF_RRI_:HF_RRI_ and LF_sys_ showed a significant correlation with self-reported anxiety over three time points, though this correlation was small and unlikely to be of clinical significance. These findings suggest that spectral analysis of cardiovascular assessments may not be a valid surrogate measure for subjective stress in all contexts.

The small, cross-sectional correlation is contrary to our initial hypothesis and earlier work by others that have reported associations between anxiety and HRV. A meta-analysis by Chalmers et al. (2014) reported that adults with anxiety disorders exhibited significantly reduced high-frequency heart rate variability (HF_RRI_) compared to healthy controls.^17^ However, their findings were based on only cross-sectional data pooled from clinical populations with diagnosed anxiety disorders, likely reflecting more severe anxiety symptoms than those seen in our sample. In contrast, our study involved a community-based sample with a lower severity of anxiety symptoms and evaluated cross-sectional and within-person changes over time using PROMIS anxiety measures and a standard set of heart rate and blood pressure spectral analyses, allowing for longitudinal and more comprehensive assessment of potential intervention effects. Our results add to a growing number of studies suggesting that the relationship between self-reported anxiety and HRV may not hold in non-clinical or subclinical populations.^18,19^

There may be several explanations for our null findings. First, our study population primarily consisted of healthy adults without specific anxiety disorders which may have limited the range in self-reported anxiety or autonomic tone reducing the power to observe correlations. HRV may be more sensitive to clinically elevated anxiety rather than subthreshold symptoms commonly observed in the general population.^17^ Also HRV may be more closely related to specific anxiety symptoms such as worry rather than others or specific anxiety disorders.^20^ Secondly, while reductions in self-reported anxiety were reported, physiological stress as measured by spectral analyses of heart rate and blood pressure may not show long-term changes in autonomic tone. Indeed, most research on correlations between HRV and self-reported stress are acute rather than long-term studies. Also, a 12-week training period may be insufficient to elicit long-term autonomic changes. Vagal stimulation also may only be effective for a subset of the population who have low vagal modulation with HF_RRI_ < 200ms^2^.^21^ Another possibility is that physiological changes in spectral analysis may occur through nonlinear mechanisms that may not correlate with self-reported measures.^22^ Laboratory assessment of HRV may not correlate with HRV in day-to-day life, and interindividual variability is very high.^23,24^ and may even be influenced by respiratory rate. In the present study even though we did not observe a change in resting respiratory rate between baseline, 6 weeks and 12 weeks^10^, but we cannot rule out the possibility of a shift in breathing pattern away from the HF to the LF range during the 12 weeks that could explain in a few subjects. Some researchers argue that HRV may reflect trait-like individual differences in emotional regulation capacity rather than state-level changes in psychological stress. ^25,26^ Therefore, researchers have cautioned against the over-reliance on HRV as a direct, singular measure for emotional states.^27^ The lack of correlation between subjective and other physiological stress measures is also apparent in acute stress studies. For example, Campbell and Ehlert reported a lack of correlation between an acute stress response to the Trier social stress test and various physiological measures including salivary cortisol, heart rate, blood pressure, and plasma cortisol and adrenocorticotrophic hormone.^28^ The lack of longitudinal changes may reflect that behavioral interventions, such as slow breathing, reduce stress primarily through psychological mechanisms including cognitive reappraisal and emotional regulation in the absence of changing the autonomic system. Lastly, methodological limitations including placebo (time and attention during assessments or treatments) and analysis methods may have limited our ability to see a significant correlation.

## Conclusion

This report contributes to the growing literature suggesting that subjective measures of anxiety and physiological measures of stress may not correlate. While we observed significant reductions in self-reported anxiety after 12-weeks of slow breathing, spectral analysis of HRV and BPV measures did not follow. Future studies should further investigate the mechanistic pathways underlying psychological and physiological stress and explore the potential of composite biomarkers for monitoring intervention response and clinical outcomes.

## Figures and Tables

**Table 1:** Baseline and definition of measures for anxiety, heart rate variability, and blood pressure variability (n=88)

Measure	Unit	Description	Baseline (median, lower quartile–upper quartile)
PROMIS Anxiety CAT	-	Self-report measure of anxiety symptoms using the Computerized Adaptive Testing of the Patient-Reported Outcomes Measurement Information System	56.1(53.3–59.7)
Root mean square of successive differences in RR intervals (RMSSD)	ms	A common time-domain measure of parasympathetic modulation.	38 (24–51)
Low frequency of RR interval variability (LF_RRI_)	ms2	Heart rate low-frequency power (0.04–0.15 Hz) of RR interval variability. Reflects a mix of sympathetic and parasympathetic modulation.	544 (319–1018)
High frequency of RR interval variability (HF_RRI_)	ms2	Heart rate high-frequency power (0.15–0.40 Hz) of RR interval variability. Primarily reflects parasympathetic modulation.	287(148–761)
Ratio of low to high frequency RR interval variability (LF_RRI_/HF_RRI_)	%	This ratio increases under well-defined condition of high sympathetic activation and vagal withdraw (like orthostatic stress). It should be used with caution during resting conditions.	1.76 (0.75–3.16)
Low frequency variability in systolic blood pressure (LF_sys_)	mmHg2	Reflects sympathetic modulation of vasomotor tone.	5.9(3.3–8.9)

**Table 2:** Partial Correlation Controlling for Age, Gender and BMI Based on Linear Mixed Model

Correlation with PROMIS Anxiety	r	Lower 0.95	Upper 0.95	P -value
RMSSD	0.017	−0.105	0.140	0.780
LF_RRI_	−0.105	−0.226	0.017	0.091
HF_RRI_	0.054	−0.068	0.177	0.384
LF_RRI_:HF_RRI_	−0.170	−0.287	−0.053	0.005
LF_sys_	−0.136	−0.255	−0.016	0.026
